# Supplementary material for: Construction of All-in-Focus Images Assisted by Depth Sensing
Source: Sensors (Basel). 2019 Mar 22;19(6):1409. doi: 10.3390/s19061409 (PMC6470578; doi:10.3390/s19061409)
Supplement: Supplementary file 1 [file sensors-19-01409-s001.pdf]

Article

Supplementary Material

Hang Liu [1], Hengyu Li [1,*,†], Jun Luo [1], Shaorong Xie [1] and Yu Sun [1,2]

1 School of Mechatronic Engineering and Automation, Shanghai University
2 Department of Mechanical and Industrial Engineering, University of Toronto
* Correspondence: lihengyu@shu.edu.cn
† Current address: No. 149, Yanchang Road, Jing'an District, Shanghai 200072, China.

Version March 18, 2019 submitted to Journal Not Specified

Abstract: This document provides supplementary information to "Construction of all-in-focus images assisted by depth sensing".

Keywords: all-in-focus, image fusion, depth sensing

1. Website of Compared Multi-focus Image Fusion Algorithms

In the experiment, the DWT method is implemented based on O. Rockinger's image fusion toolbox http://www.metapix.de/toolbox.htm. The NSCT method is implemented based on the Nonsubsampled Contourlet Toolbox in MATLAB Central (http://cn.mathworks.com/matlabcentral/fileexchange/10049-nonsubsampled-contourlet-toolbox?s_tid=srchtitle). The NSCT-PCNN method is implemented using the code downloaded from Xiaobo Qu's homepage (http://www.quxiaobo.org/index.html) and the codes of IM and GF methods are available on Xudong Kang's homepage (http://xudongkang.weebly.com/index.html), the codes of DSIFT and DCNN methods are available on Yu Liu's homepage (http://www.escience.cn/people/liuyu1/Codes.html).

1.1. Source Images

In our experiments, 5 pairs of multi-focus images that belong to different scenes shown in Fig.S1 are utilized to compare the proposed multi-focus image fusion method and other multi-focus image fusion algorithms. The source images can be downloaded from the author's GitHub website (https://github.com/robotVisionHang).

Table S1. The quantitative assessments of the GF method reconstructs a fused image using two different base and detail layers (GF_DIFF) and the same detail layer (GF_SAME).

Scenes	Methods	Metrics					
		MI	NCIE	G	P	Y	CB
1	GF_DIFF	1.3402	0.8597	0.6946	0.9023	0.9412	0.7634
	GF_SAME	1.3625	0.8615	0.7031	0.9024	0.9499	0.7657
2	GF_DIFF	1.1674	0.8426	0.6747	0.9175	0.9431	0.7627
	GF_SAME	1.1794	0.8434	0.6828	0.9177	0.9488	0.7647
3	GF_DIFF	1.1500	0.8414	0.6998	0.9115	0.9602	0.7681
	GF_SAME	1.1645	0.8423	0.7044	0.9116	0.9650	0.7709
4	GF_DIFF	1.0978	0.8382	0.6642	0.9039	0.9500	0.7527
	GF_SAME	1.1260	0.8400	0.6741	0.9039	0.9575	0.7662
5	GF_DIFF	1.1420	0.8435	0.6594	0.8953	0.9419	0.7607
	GF_SAME	1.1602	0.8447	0.6659	0.8955	0.9462	0.7638

© 2019 by the authors. Submitted to *Journal Not Specified* for possible open access publication under the terms and conditions of the Creative Commons Attribution (CC BY) license (http://creativecommons.org/licenses/by/4.0/).

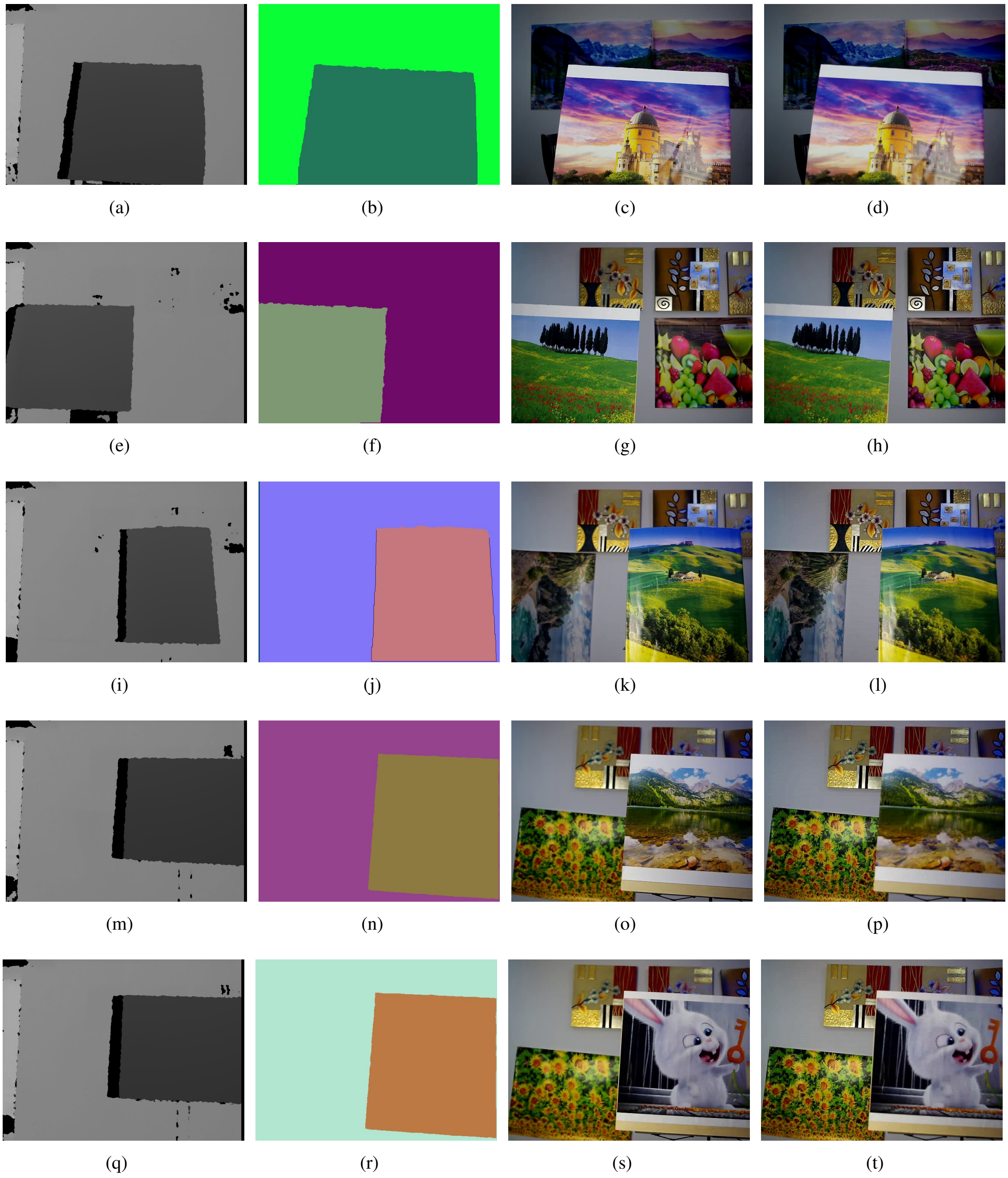

Figure S1. Multi-focus source images captured at five different scenes, the four images in each row belong to a same scene. In each row, the first image is the depth map of the scene, the second image is the segmentation result of the depth map, the front object is in best focus in the third image and the background objects is in best focus in the last image, (a)-(d) belong to the first scene, (e)-(h) belong to the second scene, (i)-(l) belong to the third scene, (m)-(p) belong to the fourth scene, (q)-(t) belong to the fifth scene.

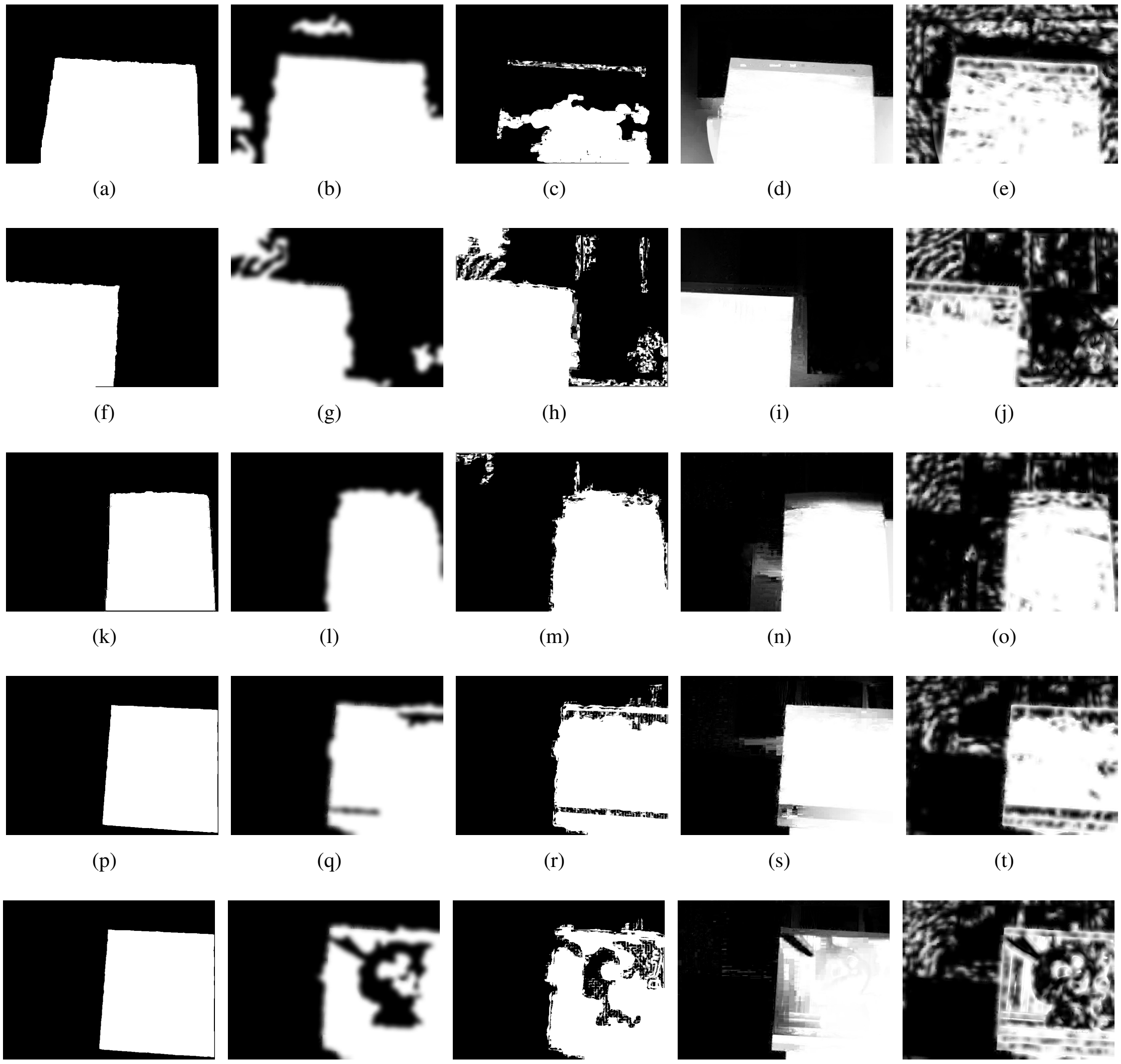

(a) (b) (c) (d) (e)

(f) (g) (h) (i) (j)

(k) (l) (m) (n) (o)

(p) (q) (r) (s) (t)

(u) (v) (w) (x) (y)

Figure S2. Weight maps generated by different methods when fusing different groups of multi-focus source images in Fig. S1. In each row, the weight map from left to right is generated by the proposed method, DCNN, DSIFT, IM and GF respectively. (a)-(e) belong to the first scene, (f)-(j) belong to the second scene, (k)-(o) belong to the third scene, (p)-(t) belong to the fourth scene, (u)-(y) belong to the fifth scene.
